# Osteosarcopenia and Pain: Do We Have a Way Out?

**DOI:** 10.3390/biomedicines11051285

**Published:** 2023-04-26

**Authors:** Roberto Bonanni, Sonia Gino Grillo, Ida Cariati, Lucia Tranquillo, Riccardo Iundusi, Elena Gasbarra, Virginia Tancredi, Umberto Tarantino

**Affiliations:** 1 Department of Clinical Sciences and Translational Medicine, “Tor Vergata” University of Rome, Via Montpellier 1, 00133 Rome, Italy; roberto.bonanni1288@gmail.com (R.B.); umberto.tarantino@uniroma2.it (U.T.); 2 Department of Orthopaedics and Traumatology, “Policlinico Tor Vergata” Foundation, Viale Oxford 81, 00133 Rome, Italy; s.ginogrillo@gmail.com (S.G.G.); tranquillolucia@gmail.com (L.T.); riccardo.iundusi@uniroma2.it (R.I.); gasbarra@med.uniroma2.it (E.G.); 3 Department of Systems Medicine, “Tor Vergata” University of Rome, Via Montpellier 1, 00133 Rome, Italy; tancredi@uniroma2.it; 4 Centre of Space Bio-Medicine, “Tor Vergata” University of Rome, Via Montpellier 1, 00133 Rome, Italy

**Keywords:** osteosarcopenia, pain, musculoskeletal diseases, anti-osteoporotic drugs, pain neurophysiology education programme, physical exercise, nutrition, interdisciplinarity

## Abstract

Osteosarcopenia (OSP) is a geriatric syndrome characterized by the coexistence of osteoporosis and sarcopenia and associated with an increased risk of fragility fractures, disability, and mortality. For patients with this syndrome, musculoskeletal pain represents the most significant challenge since, in addition to limiting the individual’s functionality and promoting disability, it has a huge psychological burden involving anxiety, depression, and social withdrawal. Unfortunately, the molecular mechanisms involved in the development and persistence of pain in OSP have not yet been fully elucidated, although immune cells are known to play a key role in these processes. Indeed, they release several molecules that promote persistent inflammation and nociceptive stimulation, resulting in the gating of ion channels responsible for the generation and propagation of the noxious stimulus. The adoption of countermeasures to counteract the OSP progression and reduce the algic component appears to be necessary, providing patients with a better quality of life and greater adherence to treatment. In addition, the development of multimodal therapies, based on an interdisciplinary approach, appears to be crucial, combining the use of anti-osteoporotic drugs with an educational programme, regular physical activity, and proper nutrition to eliminate risk factors. Based on this evidence, we conducted a narrative review using the PubMed and Google Scholar search engines to summarize the current knowledge on the molecular mechanisms involved in the pain development in OSP and the potential countermeasures to be taken. The lack of studies addressing this topic highlights the need to conduct new research into the resolution of an ever-expanding social problem.

## 1. Introduction

Bone and muscle tissue represent two components of a single functional unit, the bone-muscle unit (BMU), sharing a common embryogenetic pathway and influencing each other through mechanical and biochemical stresses [1,2,3]. Indeed, muscles through contraction apply forces to bone tissue that influence the processes of bone apposition and resorption; on the other hand, bones provide the insertion sites for muscles and represent rigid levers that make body movements possible [4]. In addition to mechanical interactions, the two tissues also communicate through the secretion of numerous factors with autocrine, paracrine, or endocrine action that influence BMU health [5]. This complex communication, known as bone–muscle crosstalk, is critical to musculoskeletal health, as the absence of mechanical stress or altered expression patterns of myokines and osteokines results in functional and structural tissue impairment [6].

Several studies have shown that a significant reduction in bone mineral density (BMD) and a drastic increase in muscle atrophy occur under conditions of microgravity or prolonged mechanical unloading [7]. Such structural abnormalities are generally accompanied by an increased expression of negative regulators of bone and muscle growth, including sclerostin and myostatin [8,9], as well as a significant reduction in positive growth regulators, such as bone morphogenetic proteins (BMPs) and pentraxin 3 (PTX3) [10,11].

Interestingly, these changes also characterize the main age-related musculoskeletal disorders, such as osteoporosis and sarcopenia [12,13], as evidenced by the progressive loss of bone and muscle mass during aging and the musculoskeletal secretome alteration that favors the establishment of a chronic low-grade inflammatory component [14,15]. The coexistence of these disorders is now referred to as osteosarcopenia (OSP), a multifactorial syndrome characterized by a progressive reduction in bone strength and muscle mass and function, associated with increased falls, fractures and mortality. Unfortunately, to date there is no unambiguous diagnostic model for OSP, so the identification of this syndrome is based on the reference definitions of osteoporosis and sarcopenia [16]. Particularly, the sarcopenic condition is characterized by low muscle strength, reduced muscle quantity, or quality and poor physical performance. On the other hand, a low T-score is indicative of reduced BMD and an osteopenic/osteoporotic condition. In general, the concomitant presence of these features, together with the biochemical evaluation of bone and muscle metabolism, could confirm the diagnosis of osteosarcopenia [17]. Although OSP is a syndrome of recent introduction in the biomedical field, its prevalence in elderly individuals is well known and widely documented. Particularly, the cross-sectional study by Huo et al. showed that in a sample of 680 patients with an average age of about 80 years who underwent dual-energy X-ray absorptiometry (DXA), 37 per cent were osteosarcopenic, predominantly women, at high risk of depression and malnutrition [18], and that this percentage increased considerably in elderly people with a history of fracture [19]. The high prevalence of OSP in the elderly is certainly influenced by several risk factors, both amendable, such as sedentariness [20], diet [21], and smoking [22], and non-amendable, such as genetic determinants [23] and age [24]. Interestingly, some of these factors predispose to the onset of musculoskeletal pain and promote its persistence [25,26,27]. Indeed, although studies on the presence and intensity of pain in OSP have not yet been conducted, the algic condition is well known to occur commonly in patients with osteoporosis, mainly due to the characteristic vertebral compression fractures that promote the development of low back pain (LBP) [28]. Furthermore, the involvement of a nociceptive component in patients with sarcopenia has increasingly emerged in recent years, suggesting the existence of a correlation between musculoskeletal pain and muscle atrophy [29].

The presence of an algic component in OSP patients dramatically complicates their management, tending to discourage adherence to pharmacological and non-pharmacological therapies. In fact, in addition to the sensory component linked to nociception, pain also has a psychological component, since it is associated with anxiety, depression, loneliness, and social withdrawal [30], as well as a functional component, since pain inevitably determines fear and therefore limitation of movement, promoting physical deconditioning that predisposes to musculoskeletal pathology and pain [31].

Timely and effective management of pain in the OSP patients should be a major goal of the physician, as pain is not only a symptom of the condition, but a condition that has a significant impact on the patient’s life in all respects. The development of interdisciplinary management approaches, customized to the patient’s needs, is fundamental in favoring the achievement of a level of pain that is acceptable to the patient, improving his or her quality of life and encouraging adherence to therapy. Therefore, the aim of this narrative review of the literature was (i) to provide an up-to-date overview of the molecular processes that determine the onset and persistence of pain in patients with osteoporosis and sarcopenia and (ii) to summarize the main pharmacological and non-pharmacological management strategies used to counteract the algic condition.

## 2. Literature Search Strategy

A total of 120 articles on the pain development in OSP and potential countermeasures were included in this narrative review. The bibliographic databases MEDLINE and Google Scholar were used to select all articles of interest published between 1945 (start date) and 2023. This non-systematic search strategy was based on the use of the following combinations of medical subject headings (MeHS) and keywords: (pain) AND (musculoskeletal system) OR (osteoporosis) OR (sarcopenia) OR (osteosarcopenia) OR (bone) OR (muscle) OR (bone mineral density) OR (muscle atrophy) OR (bone mass) OR (muscle mass) OR (preventive strategies) OR (anti-osteoporotic drugs) OR (pain neurophysiology education programme) OR (physical exercise) OR (nutrition) OR (interdisciplinarity).

A comprehensive overview of the topic was provided by including results from in vitro and in vivo experimental studies, clinical studies, randomized controlled trials (RCTs), narrative reviews, systematic reviews, and meta-analyses. Relevance to the topic was initially defined by two independent researchers who analyzed all search results, while any disagreements during the article selection process were resolved by a third researcher. A further check of the selected articles was conducted by two other authors to confirm their validity and clarify any doubts. The search process was carried out on a worldwide basis, without excluding specific geographical areas or different ethnic groups. Language filters were applied to the list of results to eliminate non-English language articles.

## 3. Chronic Pain in OSP

Chronic pain is defined as persistent or recurrent pain over a period of more than three months [32]. If this pain affects musculoskeletal structures, such as bones, joints, or muscles, we speak of chronic musculoskeletal pain. This type of pain has been classified by the classification of diseases 11th revision (ICD-11) into primary, i.e., pain that is not justified by a specific pathology of the musculoskeletal system, and secondary, triggered by underlying pathologies [33]. Primary chronic musculoskeletal pain includes pathologies such as fibromyalgia and non-specific LBP. In contrast, secondary chronic musculoskeletal pain may depend on a persistent inflammatory state, as in infections or autoimmune or auto-inflammatory diseases, on structural changes, including musculoskeletal injuries or osteoarthritis, or on diseases of the nervous system, as in Parkinson’s disease or multiple sclerosis. Therefore, diseases that lead to profound alterations of musculoskeletal structures may generate secondary chronic musculoskeletal pain due to the involvement of numerous substances that promote nociception and sensitization [33]. In this context, thoracic and lumbar vertebral fractures occurring in patients with osteoporosis are primarily responsible for LBP afflicting such patients [34]. Furthermore, Iwahashi and colleagues recently reported that sarcopenia significantly affects LBP, suggesting the existence of a correlation between muscle atrophy and nociception [35].

### 3.1. The Ion Channels of Pain

**Figure 1:**
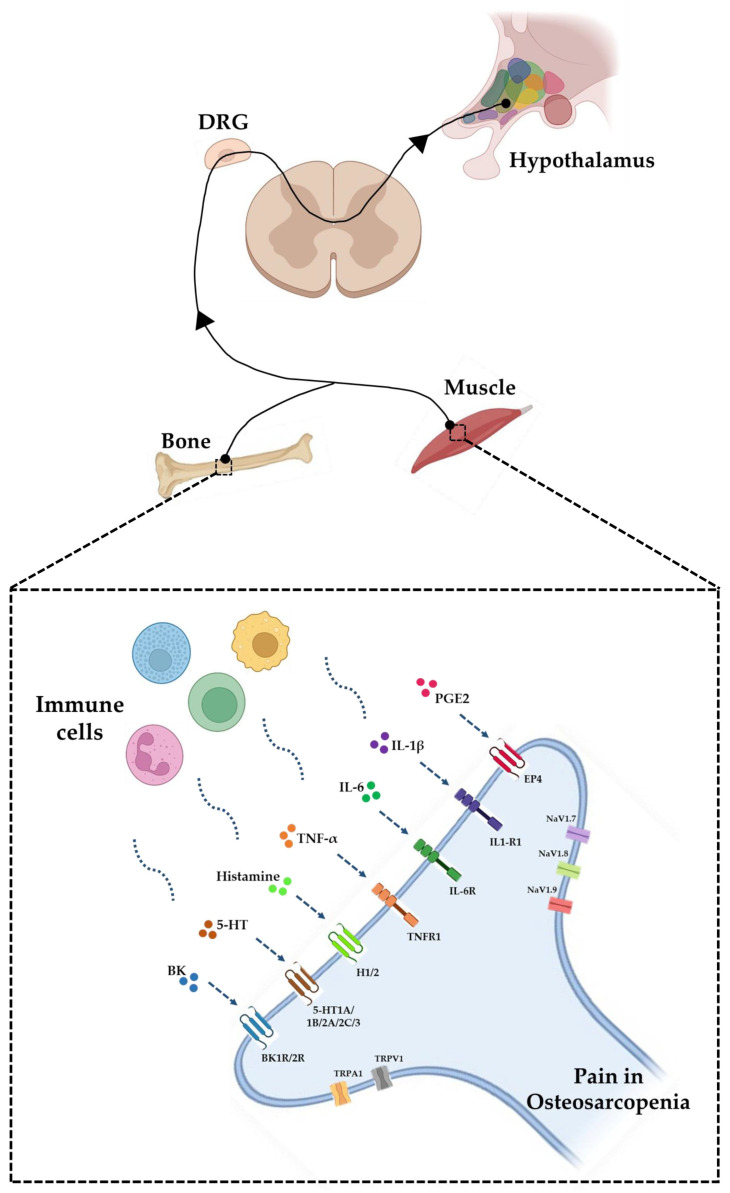
Development and transmission of the nociceptive signal in the presence of bone and/or muscle damage. The neurons responsible for encoding and transducing harmful musculoskeletal stimuli are in the dorsal root ganglia (DRG). In the presence of bone and/or muscle tissue damage, immune cells release numerous mediators, including lipid mediators such as prostaglandin E2 (PGE2), cytokines such as interleukin-1β (IL-1β), interleukin-6 (IL-6) and tumor necrosis factor α (TNF-α), as well as neurotransmitters such as histamine, serotonin (5-HT) and bradykinin (BK). All these binds to their receptors on the membrane of the nociceptive axon terminal, inducing the opening of ion channels, including transient receptor potential cation channel subfamily V member 1 (TRPV1), transient receptor ankyrin 1 (TRPA1) and voltage-dependent sodium channels (NaV1.7, NaV1.8 and NaV1.9). The resulting flux of ions promotes depolarization of the axon terminal of the nociceptive fibre, promoting the generation and transmission of the nociceptive signal.

The neurons responsible for the encoding and transduction of musculoskeletal noxious stimuli are in the dorsal root ganglia (DRG) and have a peripheral branch, whose endings deeply innervate musculoskeletal structures (Figure 1), and a central branch that establishes synapses with second-order nociceptive neurons, entering the central nervous system [36]. It is noteworthy that peripheral endings possess ligand-dependent and voltage-dependent ion channels that open in response to specific chemical signals, generating action potentials that propagate within milliseconds [37]. Among these, the transient receptor potential cation channel subfamily V member 1 (TRPV1), also known as capsaicin receptor and vanilloid receptor 1, is known to respond to stimuli of various kinds, such as high temperature, capsaicin, or low extracellular pH [38]. Phosphorylation events on the N-terminal site of TRPV1 promote positive regulation of the receptor [39]. These phosphorylations can be operated directly by specific intracellular protein kinases, such as calcium- and calmodulin-dependent protein kinase II (CaMK II kinase), or indirectly through the action of various substances, such as prostaglandins, leukotrienes, and bradykinin, which activate intracellular kinases [40]. Although the TRPV1 involvement in bone pain has long been known, few studies have investigated the role of this receptor in osteoporotic pain. In this context, Yoshino et al. found a significant increase in TRPV1-immunoreactive DRG neurons innervating the left femur of ovariectomized female Sprague Dawley rats compared to control groups, suggesting an increased TRPV1-mediated sensory transmission in osteoporotic bone [41]. In this regard, capsaicin-based therapies have been suggested to be remarkably effective in reducing skeletal pain, confirming the direct involvement of TRPV1 in bone pain [42].

The transient receptor ankyrin 1 (TRPA1) ion channel, expressed by a subpopulation of TRPV1-expressing nociceptive nerve fibers, contributes to the transduction of noxious stimuli into electrical signals and, therefore, to the onset of pain and the transition of acute pain into chronic pain [43]. The TRPA1 involvement in osteoporosis pain was suggested by Ibe et al., who investigated the relationship between changes in the bone structure of mice subjected to limb unloading and hypersensitivity to pain and cold stimulation by evaluating the effects of TRPV1 and TRPA1 antagonists [44]. An increased susceptibility to pain and cold in mice with limb suspension was observed, concomitant with a significant impairment of bone microarchitecture. It is noteworthy that the TRPA1 antagonist significantly improved cold sensitivity, suggesting a role for TRPA1 in osteoporosis bone pain [44].

Unfortunately, the role of TRPV1 and TRPA1 in pain associated with muscle mass loss is unclear. However, capsaicin has recently been reported to counteract muscle atrophy, improve cell survival, and reduce the expression of apoptotic markers, suggesting the existence of a correlation between TRPV1, muscle atrophy, and pain [45]. Furthermore, the TRPA1 involvement in the onset of spontaneous masseter muscle pain under inflammatory conditions has been proposed, suggesting a role in skeletal muscle pain [46].

In the context of chronic musculoskeletal pain syndromes, voltage-dependent sodium channels (NaV) play a key role in the excitability of nociceptive neurons, as genetic variants of these channels have been associated with chronic pain disorders [47]. Of these, the NaV1.7, NaV1.8, and NaV1.9 subtypes are abundantly expressed in DRG sensory neurons, where they amplify small subthreshold depolarizations by preparing the nociceptor to act as a threshold channel regulating excitability [37,48]. NaV are known to play a role in inflammatory pain, as blockers of these channels, such as local anesthetics or tricyclic antidepressants, may have therapeutic potential in pain management [49]. These are expressed on muscle nerve afferents. Particularly, NaV1.8 and NaV1.9, which are extensively involved in nociception and chronic pain, are abundantly expressed in small- and medium-caliber sensory neurons where they mediate the exercise pressure reflex [50]. The chemical signal responsible for the activation of these ion channels comes from resident immune cells in the tissue, which release specific mediators of various kinds that, through binding to their receptor on the nociceptor membrane, induce intracellular responses that culminate in pain perception [51].

### 3.2. The Crosstalk between the Immune System and the Nervous System

In the presence of tissue damage, immune cells release numerous chemical mediators that bind to their receptors on the membrane of the nociceptive terminal. Of these, lipid mediators are undoubtedly the ones most involved in pain in musculoskeletal structures. For example, the release of prostaglandin E2 (PGE2) by neutrophils and macrophages and binding to the EP4 receptor, in conjunction with TRPV1 signaling, is known to be associated with the transition of acute pain into chronic pain [52]. However, PGE2 has been observed to stimulate muscle stem cells in young mice, playing an important role in the regeneration of damaged muscles [53]. In this context, the increase in 15-hydroxyprostaglandin dehydrogenase (15-PGDH), the PGE2-degrading enzyme, is a hallmark of aged muscles. In fact, inhibition of this enzyme in aged mice is able to increase mitochondrial function as well as muscle mass and strength, suggesting a crucial role for PGE2 in inducing pain sensitivity and promoting reparative processes, ameliorating muscle atrophy [54]. In this context, Markworth et al. profiled the lipidome mediator of aging muscle, finding chronic muscle inflammation in aged mice caused by a marked deficiency of pro-repair mediators. In fact, although similar amounts of the pro-inflammatory cyclo-oxygenase eicosanoids, such as PGE2, were produced by young and old mice following muscle injury, only the old mice produced lower amounts of pro-resolving enzymes, including lipoxins, D-resolvins/protectins, E-resolvins, and tarsins, leading to a delay in healing and strength recovery [55].

Conflicting data have been reported on the PGE2 role in bone tissue. Particularly, PGE2 has been observed to promote osteoclastogenesis by inhibiting osteoprotegerin (OPG) production and stimulating receptor activator of the nuclear factor-kappa B (RANK) production, thus contributing to bone resorption that occurs during osteoporosis [56]. Noteworthy, an elevated concentration of PGE2 in the subchondral bone of mice produced by osteoblasts has been reported to characterize the early state of osteoarthritis prior to articular cartilage degeneration. Interestingly, capsaicin reduced the microstructural impairment of subchondral bone architecture, as well as joint degeneration and pain, confirming the role of TRPV1 in the musculoskeletal pain development [57]. Although some evidence supports an important role of PGE2 in musculoskeletal tissue repair, an association between PGE2 expression and poor bone and muscle quality has also been found, suggesting its involvement in the nociception of osteoporosis and sarcopenia [58].

Several cytokine factors, such as interleukin-1β (IL-1β), interleukin-6 (IL-6) and tumor necrosis factor α (TNF-α), are also responsible for nociceptive stimulation to musculoskeletal structures, playing a key role in pain sensitivity [59]. Indeed, DRG nociceptors express IL-1β receptors (IL-1R) and IL-6 receptors (IL-6R) and these signaling are associated with increased pain sensitivity [60,61]. Furthermore, IL-6 and TNF-α are known to up-regulate inflammatory pathways that lead to muscle cell aging and skeletal muscle deterioration [62]. In this context, Bian and colleagues investigated the levels of IL-6 and TNF-α in 441 elderly people divided into two groups, depending on the presence or absence of sarcopenia diagnosed with the criteria set by the European working group on sarcopenia in older people (EWGSOP) and the Asian working group for sarcopenia (AWGS) [63]. Interestingly, a significant difference in bone mineral content (BMC) between sarcopenic and non-sarcopenic patients was found, highlighting the correlation between muscle and bone tissue quality. Noteworthy, serum levels of IL-6 and TNF-α were significantly higher in sarcopenic patients, suggesting the role of these cytokines as inflammatory predictors of sarcopenia [63]. Similarly, Rong et al. investigated IL-6 and IL-10 levels in 164 elderly people, divided into a control group and a sarcopenic group, and found a significant increase in the IL-6/interleukin-10 (IL-10) ratio [64]. In relation to osteoporosis, a correlation between IL-6 and skeletal pain was also observed, as the use of IL-6 inhibitors reduced pain in ovariectomized mice, although not restoring normal bone structure [65]. Similar results were found in mice undergoing limb unloading, as the use of IL-6 inhibitors restored the nociceptive threshold but did not counteract bone loss [66]. Noteworthy, Liu et al. observed that PGE2, in addition to stimulating osteoclast differentiation by suppressing OPG, also promotes IL-6 production, suggesting the existence of an interactive and synergistic effect of PGE2 and IL-6 signaling in osteoclastogenesis [56].

Finally, certain neurotransmitters such as histamine, serotonin (5-HT) and bradykinin (BK), which are released by immune cells, would appear to favor the onset and persistence of musculoskeletal pain, as well as the expression of specific NaV and TRPV1 reactivity [67,68,69]. Interestingly, Uchitomi and colleagues conducted a metabolomics analysis of skeletal muscle from 8- and 28-month-old mice by time-of-flight mass spectroscopy with capillary electrophoresis, finding significant differences in metabolites between the two experimental groups. Particularly, a significant increase in the histamine and serotonin, 2.6- and 1.7-fold, respectively, was found in the gastrocnemius muscle of the aged mice, probably due to the muscle lesions associated with aging [70]. In this context, mast cells, immune cells that release histamine and serotonin affecting bone metabolism, have been proposed to promote osteoclast formation by participating in the osteoporosis pathogenesis. In fact, histamine-deficient mice show increased bone mass, being protected from the bone degeneration that occurs following ovariectomy [71]. In agreement, blocking the histamine receptor in ovariectomized rats prevents bone loss by reducing the number of osteoclasts [72,73].

In summary, the tissue damage that characterizes osteoporosis and sarcopenia is followed by the release of numerous mediators by the cells of the immune system, which bind to their receptors on the membrane of the nociceptive axon terminal, inducing the opening of ion channels and promoting depolarization of the axon terminal of the nociceptive fiber, culminating in the generation and transmission of the nociceptive signal. Changes in the membrane potential of peripheral nerve terminals cause the release of vesicles containing neuropeptides, such as calcitonin gene related peptide (CGRP) and substance P (SP), with potent effects on vasodilation, promoting further influx of immune cells to the site of injury. The degenerative nature of osteoporosis and sarcopenia, together with the chronic low-grade inflammation that characterizes the elderly, could feed this circuit by promoting pain persistence [74]. Indeed, although nociceptive neurons in the somatosensory nervous system are neurons with a high activation threshold, they also undergo plastic changes and may lower their activation threshold. This mechanism of neuroplasticity is called peripheral sensitization, a form of synaptic plasticity that, by lowering the activation threshold of nociceptors, protects the injured tissue [75]. However, in the case of prolonged nociceptive stimulation, nociceptive pathways may undergo changes in increased function and response, leading to neuronal changes at the central nervous system level, a phenomenon known as central sensitization [76]. Therefore, the medical management of OSP patients with musculoskeletal pain should strongly consider this aspect and ensure strict adherence to therapy by the patient to counteract the persistence of pain.

## 4. Management of Pain in OSP Patients

**Figure 2:**
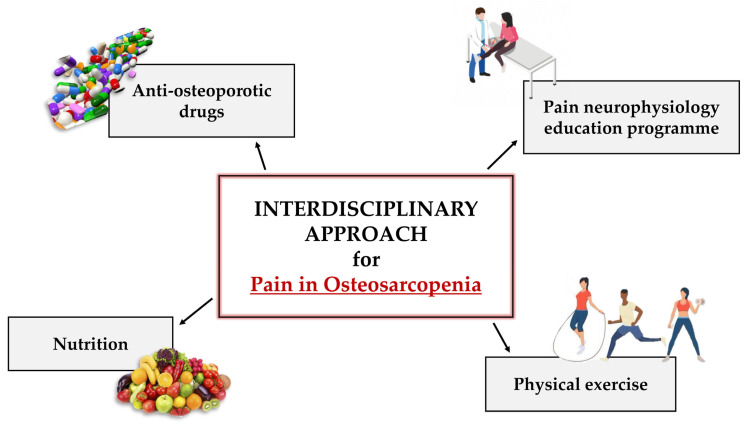
Interdisciplinary approach for the management of osteosarcopenic (OSP) patients. The pain management in OSP patients should involve an interdisciplinary approach, in which several professionals collaborate in the development of a therapy aimed at minimizing the pain perception. This strategy should include both multimodal pharmacological therapies, based on the use of anti-osteoporotic drugs, and non-pharmacological therapies including pain neurophysiology education programme (PNE), physical exercise and nutrition. Such an integrated approach will be essential to act simultaneously on the algic and musculoskeletal components.

The management of musculoskeletal pain in OSP patients requires an interdisciplinary approach in which multiple professionals collaborate in the development of a therapy aimed at reducing pain to a minimum, reaching a level appropriate to the patient. In this context, a fundamental role should be played by musculoskeletal tissue specialists since, although action on the algic condition is a priority, improvement of bone and muscle quality is equally necessary to counteract the OSP progression. Therefore, the development of an integrated approach, based on the use of a multimodal pharmacological therapy and a non-pharmacological therapy, is fundamental to act simultaneously on both the algic and the musculoskeletal component (Figure 2).

### 4.1. Pharmacological Therapy: The Power of Anti-Osteoporotic Drugs

Several pharmacological solutions are currently in use to alleviate musculoskeletal pain. Of these, non-steroidal anti-inflammatory drugs (NSAIDs) and opioids are known to reduce pain, although their efficacy is limited to the short term and the associated adverse effects force careful consideration [77]. Paracetamol is often indicated as initial therapy and its high safety favors even long-term administration [78]. Therefore, the development of an effective, safe, and long-term pharmacotherapy that can considerably reduce pain is a complex issue and requires the participation of all specialists in the interdisciplinary team. On the other hand, it is important to consider that some of the drugs used in anti-osteoporotic therapy have a surprising anti-nociceptive effect, as numerous trials have demonstrated their ability to reduce patient-perceived pain and improve quality of life. Importantly, although there is still no specific pharmacotherapy for sarcopenia, some anti-osteoporotic drugs have recently been reported to also improve muscle tissue quality, suggesting the possibility of developing targeted therapies for OSP patients with chronic musculoskeletal pain.

#### 4.1.1. Bisphosphonates

Bisphosphonates are considered first-line drugs in the osteoporosis treatment, as their high affinity for the bone mineral matrix allows them to reduce bone turnover by inhibiting osteoclastic activity [79]. Interestingly, their discrete analgesic power has recently been proposed, as demonstrated by the reduction in chronic LBP, due to vertebral fractures or subsidence, in OSP patients [80]. Specifically, Ohtori et al. conducted an observational study by administering oral risedronate 2.5 mg/day for 4 months to 27 post-menopausal women with LBP without vertebral fractures [81]. Changes in BMD were assessed in association with changes in pain measured by visual analogue scale (VAS) and in urinary and serum levels of N-terminal telopeptide of collagen type I (NT-x). Noteworthy, a significant improvement in LBP was found after 4 months of risedronate treatment, in association with a marked reduction in serum and urinary NT-x, suggesting that bone resorption can cause LBP even in the absence of osteoporosis and underlining the efficacy of risedronate in counteracting the algic condition [81]. Similarly, Iwamoto and colleagues conducted a RCT to compare the effects of elcatonin, a calcitonin analogue used for its anti-nociceptive effects, and alendronate in 194 post-menopausal women with osteoporosis and LBP [82]. The patients were randomized into two groups, one receiving oral treatment with 35 mg/week of alendronate and the other treated with 20 U/week of intramuscular elcatonin. Interestingly, a rapid analgesic effect of alendronate was observed, as demonstrated by the significant improvement in LBP and quality of life compared to patients treated with elcatonin [82].

The action of bisphosphonates may not be limited to bone tissue, as a beneficial action of zoledronate on skeletal muscle has recently been suggested. Particularly, Huang et al. conducted a retrospective case-control cohort study to investigate the efficacy of zoledronic acid monotherapy on the muscle mass of osteoporotic patients. Surprisingly, a significant increase in muscle mass was observed in patients who received zoledronic acid treatment for three years, as opposed to the control group who suffered further muscle loss, suggesting the possibility of using zoledronic acid to counteract the OSP progression [83]. Overall, bisphosphonates could represent a class of drugs with anti-nociceptive power that can preserve bone and muscle mass in patients with osteoporosis and sarcopenia. Although BMD preservation has been a known effect for some time, further studies investigating the efficacy of various bisphosphonates on both bone mass and improvement of the algic condition appear to be necessary to develop appropriate therapies for osteosarcopenic patients with chronic musculoskeletal pain.

#### 4.1.2. Denosumab

Denosumab is a monoclonal antibody directed against the RANK ligand (RANKL) protein secreted by osteocytes that, through binding to the RANK receptor, stimulates osteoclast proliferation, promoting bone resorption [84]. Subcutaneous injection of denosumab is known to minimize markers of bone resorption, promoting significant protection from vertebral and appendicular fractures [85]. The analgesic efficacy of denosumab emerged in the retrospective single-center study by Tetsunaga et al., who analyzed the changes in BMD and pain perception of 80 patients with osteoporotic vertebral fractures treated with 60 mg denosumab subcutaneously every six months or with 35 mg per week of alendronate administered subcutaneously [86]. Regarding the effect on BMD, a significant improvement was observed in patients treated with denosumab rather than alendronate. About pain score, the two drugs showed an overlapping efficacy profile, although denosumab produced significantly better pain relief than bisphosphonate as early as the second week of treatment [86]. Similarly, Petranova and colleagues evaluated the efficacy and safety of treatment with 60 mg subcutaneous denosumab in post-menopausal women with or without concomitant glucocorticoid therapy. In both groups, denosumab treatment produced an increase in BMD and a significant reduction in pain, confirming the efficacy and safety of the drug for both counteracting osteoporosis and relieving pain symptoms [87]. Interestingly, new evidence in favor of denosumab’s efficacy on muscle mass has emerged in recent years, including the study conducted by Bonnet et al., in which an increase in appendicular lean mass and hand grip strength in post-menopausal women undergoing anti-osteoporotic therapy with denosumab for 3 years was found concomitantly with an increase in BMD [88]. Finally, Aryana and colleagues recently demonstrated a greater efficacy of denosumab compared to placebo and bisphosphonates in improving muscle mass and reducing the risk of falls, suggesting a role for OSP treatment [89].

#### 4.1.3. Teriparatide

Teriparatide, human parathyroid hormone (PTH) 1–34, is an anabolic drug for osteoporosis, known to prevent the occurrence of new vertebral fractures and counteract LBP. The analgesic efficacy of this anabolic drug was highlighted in Chen et al.’s retrospective cohort study of 112 postmenopausal women with LBP caused by osteoporotic vertebral compression fractures. Specifically, women treated with 20 μg subcutaneous teriparatide once daily for 12 months showed a greater reduction in pain, as assessed by VAS, than control women treated with calcium and vitamin D alone [90]. In agreement, Ifuku and colleagues conducted an observational study of 3573 patients with osteoporosis to evaluate the efficacy of teriparatide, administered once a week for 72 weeks, in counteracting the osteoporotic condition and the occurrence of fractures. Noteworthy, a significant reduction in VAS scores for LBP as early as week 24 was observed, in association with a significant increase in femoral BMD and lumbar spine BMD, revealing an improvement throughout the administration period [91]. The analgesic action of teriparatide was also confirmed by Kato et al., who subjected a group of mice to ovariectomy and treatment with the anabolic drug, evaluating bone microarchitecture, pain, and the expression of TRPV1 and CGRP in DRG neurons. In contrast to what was observed in control mice, subcutaneous injections of teriparatide significantly reduced the mechanical hyperalgesia and expression of TRPV1 and CGRP induced by ovariectomy, as well as preventing bone loss, highlighting the analgesic efficacy of the drug [92]. Interestingly, beneficial effects of teriparatide have also recently been observed on skeletal muscle, suggesting it as a potential candidate for the OSP treatment. Specifically, Sato et al. subjected female Wistar rats to ovariectomy and tail suspension to evaluate the efficacy of both teriparatide and treadmill exercise. Surprisingly, a significant improvement in BMD and skeletal muscle mass was observed in the teriparatide-treated group of rats, like those subjected to exercise, while the percentage of fat mass and adipose tissue in the bone marrow was markedly reduced [93]. More recently, Fujimaki and colleagues studied the effect of PTH on muscular atrophy and dysfunction in 8-week-old female rats undergoing ovariectomy and found a significant improvement in motor capacity, as assessed by grip strength and maximum running speed, and muscle weakness, in association with the bone mass preservation [94].

### 4.2. Non-Pharmacological Therapy: From Mind to Body

Numerous non-pharmacological approaches can be taken to improve musculoskeletal pain. However, pain is not only a sensory experience for the patient, but also a psychological experience with a significant impact on mental health. Therefore, an ideal management approach should not only address physical well-being, by eliminating those risk factors that predispose to inflammation and musculoskeletal pain, but also mental well-being, to provide the patient with appropriate psychological support and tools for self-management. Among these, a pain neurophysiology education programme (PNE), regular physical activity and a proper diet are undoubtedly the most frequently used non-pharmacological therapeutic strategies to combat musculoskeletal pain.

#### 4.2.1. PNE

In 2011, Louw et al. conducted a systematic literature search to evaluate the evidence for the PNE effectiveness in improving disability, anxiety and stress associated with chronic musculoskeletal pain. Eight high quality RCTs, one comparative study and one pseudo-RCT were selected, gathering sufficient evidence to support PNE as an educational strategy to reduce chronic musculoskeletal pain, disability and promote movement by improving physical performance [95]. However, in the same year, Clarke and colleagues showed the results of a systematic review with meta-analysis of RCTs to evaluate the evidence supporting PNE in pain management in patients with chronic LBP. According to the authors’ conclusions, PNE produces statistically significant improvements in pain, but clinically small to justify its use in the management of patients with chronic LBP [96]. More recently, Saraçoğlu et al. subjected patients operated for lumbar radiculopathy to a 70-min pre-operative PNE programme to reduce post-operative pain, kinesiophobia and disability [97]. It involved sessions, led by a certified PNE expert physician, consisting of 70 min of face-to-face time during which metaphors, anecdotes and images were used. Although statistically significant reductions in LBP and leg pain were not found, significant improvements in kinesiophobia and disability were observed in patients who had adhered to the PNE programme [97]. The contradictory results regarding the PNE effectiveness in counteracting musculoskeletal pain suggest the need to further investigate the usefulness of this practice in pain management. Indeed, the PNE programme administration should be preceded by a careful clinical biopsychosocial assessment of the patient to allow an adequate explanation of the mechanisms underlying the onset and persistence of pain. However, the absence of clear guidelines on how to perform a biopsychosocial assessment makes it difficult to classify patients specifically and consequently to administer an appropriate PNE programme that is useful for the patient with musculoskeletal pain [98].

Noteworthy, PNE proved to be most effective when administered in combination with exercise programmes, suggesting that a non-pharmacological therapy based on the combination of PNE, and exercise can provide a statistically and clinically significant reduction in pain. In this context, Bodes Pardo and colleagues randomized 56 patients with chronic LBP into two groups, a therapeutic exercise group that was administered a multimodal exercise programme consisting of stretching and aerobic exercise, and an intervention group that underwent the same multimodal therapeutic exercise programme in combination with a PNE programme [99]. Specifically, the PNE programme consisted of two sessions of 30–50 min each, characterized by verbal explanation and visual representation of all the main concepts of pain neurophysiology. Pain, which was comparable between the two patient groups at baseline, was significantly reduced in both treatment groups and this reduction continued until the third month of follow-up. However, the intervention group showed a greater reduction in pain than the exercise group, highlighting the extraordinary efficacy of this combination therapy on chronic LBP [99]. In agreement, Miller et al. investigated the effectiveness of a PNE programme administered in combination with exercise in patients with chronic non-oncological pain, finding significant improvements in function and musculoskeletal pain compared to usual care [100]. Specifically, the patients underwent two weekly visits for 6 weeks, a 1.5-h group visit aimed at education, self-management and pain science, and cognitive-behavioral principles to support behavior change. The second visit lasted 30–45 min and was individual in nature, aimed at the implementation of self-management plans and the development of personalized exercise programmes [100]. Therefore, an appropriate PNE programme could not only provide the motivation the patient needs to adhere strictly to drug therapy, but also counteract sedentariness by optimizing the beneficial effects of exercise.

#### 4.2.2. Physical Exercise

The beneficial effects of exercise on musculoskeletal health, as well as on the algic condition, are well known and widely documented. In fact, constant exercise, in addition to being the best strategy for preventing OSP and associated pain, is also a valuable ally in counteracting the progression of these conditions [101,102]. In this regard, exercise has been reported as the only strategy to consistently prevent fragility and improve sarcopenic condition and physical function in the elderly, increasing muscle strength, aerobic capacity and endurance in the elderly [103]. In relation to bone quality, exercise is known to result in an increase in specific bone density sites, particularly the femoral neck and lumbar spine, proving to be a good ally for the management of OSP patients [104]. The effectiveness of this non-pharmacological strategy on musculoskeletal health lies in the molecular mechanisms underlying exercise, as the expression of some important regulators of bone and muscle growth is known to be influenced by levels of physical activity [105]. For example, regular exercise can modulate the expression of myostatin, the main negative regulator of muscle growth, and irisin, a myokine with osteoinductive power, positively influencing musculoskeletal health [106,107]. Importantly, similar effects can also be achieved in sedentary patients using vibratory platforms, known to be a valid alternative strategy to exercise [108,109,110].

The benefits of exercise are not limited to the musculoskeletal system but affect the entire organism. Particularly, post-workout FNDC5/irisin release has been reported to promote the expression of brain-derived neurotrophic factor (BDNF), a neurotrophin that markedly reduces the risk of depression [111]. Noteworthy, depression is a risk factor closely associated with pain as between 30 and 60 per cent of people with chronic pain also suffer from depression and this association dramatically worsens the patient’s quality of life. Therefore, exercise, in addition to benefiting the structure and biochemistry of the musculoskeletal system, could also reduce the depressive state of pain patients and promote adherence to treatment [112].

#### 4.2.3. Nutrition

Several studies agree that regular and appropriate exercise should be associated with a healthy and balanced diet. Indeed, poor diet is a predictor, perpetuator, and underlying factor of musculoskeletal pain, promoting inflammation and the risk of obesity [113]. In general, a diet rich in saturated fatty acids and sugars is known to increase serum levels of pro-inflammatory cytokines, while omega-3-rich and plant-based foods have an antioxidant and anti-inflammatory effect, reducing musculoskeletal pain [114]. In this context, Perna et al. conducted a systematic review of RCTs, cross-sectional studies and observational studies concerning the use of nutritional and antioxidant interventions to counter pain in adults with musculoskeletal disorders. Dietary advice aimed at outlining the most suitable foods to be included in the daily diet has proved particularly useful, indicating a weekly consumption of fish and omega-3 fatty acids, as well as supplementation with milk, vitamin D and magnesium, if necessary [115]. Furthermore, the use of whey protein-containing supplements in combination with an endurance exercise programme has been proposed as a valid strategy to promote increased femoral and vertebral BMD in elderly men with OSP, highlighting the ability of such supplements to prevent the risk of fracture events [102]. Vitamin D supplementation is also a key countermeasure for OSP patients, both for its effect on bone tissue and for its anti-inflammatory and analgesic properties. In fact, supplementation with 5000 IU vitamin D per week for three months significantly reduced pain symptoms and improved quality of life. The action of vitamin D also extends to skeletal muscle, as it is crucial for muscle strength, function, and coordination [116,117]. The effectiveness of vitamin D in reducing pain in musculoskeletal disorders was highlighted by Wu et al. who conducted a systematic review including 19 RCTs and 3436 participants. A statistically significant reduction in pain scores in patients who had received vitamin D supplementation compared to placebo was observed, hypothesizing an important role for such supplementation in the chronic musculoskeletal pain management [118]. These observations were recently confirmed by the systematic review of Lombardo and colleagues, who demonstrated that vitamin D supplementation in patients with established deficiency produced significant improvements in chronic widespread pain [119]. Surprisingly, the analgesic effect of vitamin D was correlated with its anti-inflammatory properties, which result in a reduced release of cytokines and PGE2, suggesting its supplementation as an obligatory step in OSP patients [120].

Overall, an adequate non-pharmacological therapy should include an appropriate physical exercise programme aimed at strengthening both body and mind, preparing OSP patients to face the complex challenge against musculoskeletal pain. Such a strategy should be supported by an optimal nutritional plan, to reduce inflammation and body weight, favoring the functionality of the musculoskeletal system and reducing hyperalgesia.

## 5. Conclusions

OSP is a multi-system geriatric disease that is reaching epidemic proportions, due to the constant and progressive aging of the population and the sedentary lifestyle. The impairment of bone microarchitecture and muscle mass predispose individuals with this syndrome to fragility fractures and the subsequent onset of pain. Moreover, the constant chronic inflammation typical of elderly individuals favors nociceptive stimulation, promoting pain sensitization and its persistence due to prolonged stimulation. Particularly, the release of PGE2, IL-6 and histamine by immune cells initiates intracellular changes that result in gating of ion channels and propagation of the nociceptive signal.

Although it is important to preserve musculoskeletal structure and function, as well as to counteract the OSP progression, it must be kept in mind that musculoskeletal pain is the leading cause of disability in these patients, suggesting the need to develop strategies to counteract the algic condition. However, to date, there is no specific therapy for either OSP or associated pain. Therefore, the development of countermeasures capable of counteracting the progression of this musculoskeletal syndrome and simultaneously reducing the algic component appears to be necessary. Recently, some anti-osteoporotic drugs with a discrete analgesic effect have also been proposed to bring about muscular improvements, suggesting their usefulness in osteosarcopenic therapy. In addition, non-pharmacological strategies aimed at reconceptualizing pain knowledge and correcting risk factors that predispose to OSP and associated pain, such as sedentariness and poor diet, appear to be crucial. Therefore, an interdisciplinary approach in which several specialists collaborate to develop a multimodal therapy is necessary to preserve musculoskeletal health and reduce pain.

To our knowledge, this is the first up-to-date literature review that aims to highlight the molecular mechanisms involved in the pain development in OSP and potential defensive strategies to be adopted. Unfortunately, knowledge regarding the development and persistence of pain in this geriatric syndrome is still largely incomplete, highlighting the need for further studies to investigate the underlying molecular mechanisms and test the efficacy of potential innovative strategies. It is important not to resign oneself to pain because *pain is more painful if it is silent*.

## Data Availability

No new data were created or analyzed in this study. Data sharing is not applicable to this article.
